# Device for data-acquisition from transient signals: kinetic considerations

**DOI:** 10.1155/S1463924690000268

**Published:** 1990

**Authors:** A. Sanchez Sampedro, S. Sagrado Vives, J. Martinez Calatayud

**Affiliations:** Departamento de Química Analítica Facultad de Química Universitat de València Burjassot Valencia 46100 Spain

## Abstract

This paper reports on the evaluation and testing of a home-made device. Data-acquisition, treatment of transient signals and the hardware and software involved are discussed. Some practical aspects are developed in order to power the autonomy of procedures using the device. Kinetic and multi-signal calculations are considered in order to cover the actual tendencies in continuous-flow analysis. Somepractical advantages versus the use of classical chart recorders or commercial computerized-instrument devices are pointed out.


Journal of Automatic Chemistry, Vol. 12, No. 5 (September–October 1990), pp. 199–204

### Device for data-acquisition from transient signals: kinetic considerations

**A. Sanchez Sampedro, S. Sagrado Vives and J. Martinez Calatayud†**

*Departamento de Química Analítica, Facultad de Química, Universitat de València, 46100 Burjassot, Valencia, Spain*

*This paper reports on the evaluation and testing of a home-made device. Data-acquisition, treatment of transient signals and the hardware and software involved are discussed. Some practical aspects are developed in order to power the autonomy of procedures using the device. Kinetic and multi-signal calculations are considered in order to cover the actual tendencies in continuous-flow analysis. Some practical advantages versus the use of classical chart recorders or commercial computerized-instrument devices are pointed out.*

### Introduction

Automation in analytical chemistry should be considered to be the result of a suitable combination of transductors and microprocessors with an interface allowing communications between the two. A review by Betteridge and Goad [1] and a special issue of *Talanta* [2] show how automation has become incorporated into analytical chemistry. For instance, terms such as read-out device, data acquisition system, automated programmable analyser, computer-aided, computer simulation and expert system are common words in the current analytical literature. A modern well-equipped research or service laboratory needs data acquisition and instrument system control, so it is essential to introduce analytical researchers and specialists to microprocessors and addressing, accessing or interfacing concepts. The authors believe that this subject should be included in undergraduate chemistry courses.

The main reason for the increasing use of computers in analytical laboratories is their considerable advantages compared with conventional chart recorders. These advantages include: effectiveness (for example signal information in real time, data treatment during acquisition); versatility (for example the possibility of communicating, moving or saving analytical information); independence (if a total system automation is obtained); and the commercial availability of small and inexpensive microcomputers.

Flow Injection Analysis (FIA) is a consolidated analytical methodology offering reliability, versatility and speed. FIA is a suitable and inexpensive technique for handling large numbers of samples and so it is a good choice for on-line process control. A reasonable number of articles on computer applications in FIA have been published.

† To whom correspondence should be addressed.

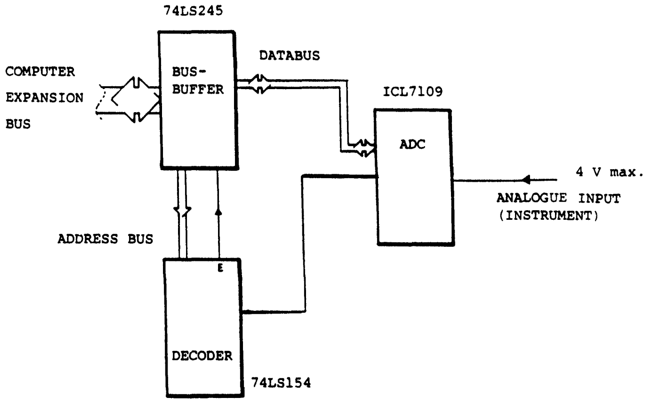

*Figure 1. Block diagram of the data acquisition device.*

Recent papers have described the present interest in flow systems process control [3]; strategies involving economic and autonomous devices [4]; and software for pattern search applications [5]. Examples of FIA-related techniques, such as HPLC, are also available [6,7]—software for simulation [8,9] and expert systems [10–12] have also been discussed.

This article describes a device made by the authors for data-acquisition and treatment of signals from different continuous flow detectors. The paper includes discussions about hardware, software and applications. Some aspects have received special attention: versatility, output, usefulness, and mathematical treatments.

The FIA analytical process is basically a kinetic one [13,14]. The software described recognizes this and was produced to provide analytical information that is not available from the current commercial computerized instrumentation.

Finally, the low cost of the home-made device (less than $40.00 for materials), as well as its simplicity, portability, ease of manipulation, and its ability to automate the whole of the assembly, are additional advantages versus the commercial instruments available.

It should be noted that the device could be adapted for a general use; the software described here, however, has been prepared and tested for the treatment of the transient signals produced by FIA or HPLC assemblies.

### Experimental section

### *Hardware*

A basic program called 'PICOS.FIA' was produced for the Amstrad CPC128 (single board, 128 K Ram and Z80 microprocessor). This computer has a relatively high power, it is easily available and the price is low (about $500).

0142–0453/90 $3.00 © 1990 Taylor & Francis Ltd.

*Device considerations*

Input operations have been performed through an on-board home-made interface shown in figure 1. The main features of the interface are: ADC (from Intersil) 12 bit binary (plus polarity and overrange), dual slope integrating analogue-to-digital converter, TTL compatible three-state outputs, 16 I/O lines. It operates up to 30 conversions.

*Software*

The BASIC program, PICOS.FIA, was originally written for the Amstrad; however, it could easily be adapted to any other computer. An important feature of the program is simplicity. Direct commands, shown on the screen, permit interactive communication between operator and computer. Any menu contains keys for accessing any section of the program. The following options are available from the main menu.

*Option 1: Information*

A short text introduces the operator into the main features of the software and gives instructions for use.

Software functions:

Situations: $t_1$, peak detected, base line value fixed.
$t_2$, start area and time-width calculation.
$t_3 = t_R$ (residence time), maximum signal value, $h_{max}$, fixed.
$t_4$, change speed acquisition, close injection valve.
$t_5$, change speed acquisition, open injection valve, end of peak, waiting next peak.

Real-time data: $t_X$ (in s) any time during acquisition (from $t_1$).
$t_X$ (in mV) signal value.
$A_X$ (in mV.s) partial area.
$S_X$ (in mV/s) slope calculated from $t_X$ and $t_{X+1}$

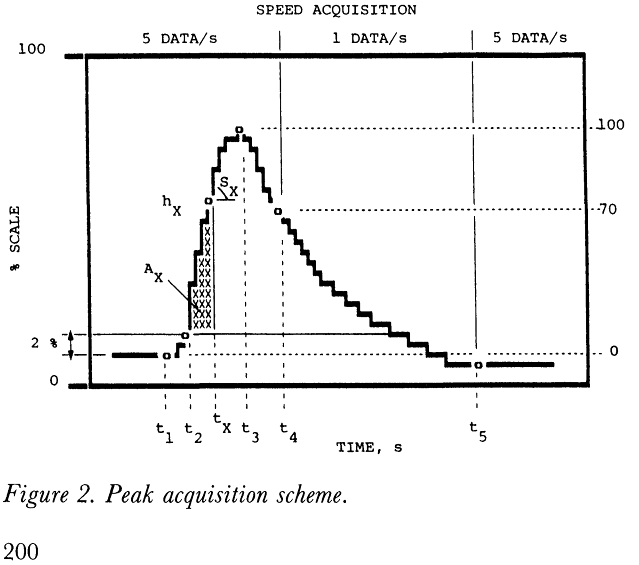

*Figure 2. Peak acquisition scheme.*

*Option 2: Start acquisition*

Option 2 offers different work possibilities: (*a*) Normal mode: for acquisition of single peaks; (*b*) Multipeak mode: for outputs producing more than one peak per injection to be considered; and (*c*) Kinetic mode: for signals requiring slope calculation. Once the required mode is selected, the output is plotted on the screen as mV versus time (min). Numerical values are divided into three different categories: (1) real time data (corresponding to the output in progress); (2) peak results (final parameters from each complete signal, depending the mode selected); and (3) series results (average results and statistical data corresponding to a desired number of consecutive injections). Peak and series results can be simultaneously printed.

The software can fix points during transient signal acquisition by comparative conditions. These points allow velocity to be changed in acquisition and they could be used to start/stop injection of a sample by means of the six-port valve in an automatic FIA assembly. Another important feature is the presentation of four real-time parameters, which are continuously shown on the screen. Figure 2 shows all these aspects, conditions and different stages during a peak acquisition.

*Option 3: Change of conditions*

Standard conditions are fixed initially by the program. This option permits the selection of some conditions: mV and min scales, conversion factor (it controls the transformation of the digital signal into mV), sensitivity in peak detection (software conditons for detecting the start and end of the peak), and statistics (number of peaks/series, and maximum relative standard deviation [RSD] for a series). The last condition implies an automatic selection of the 'correct' peaks with elimination of the 'wrong' ones (based on the limits prefixed for the preselected RSD value).

*Option 4: Disk communications*

This option permits the acquired data to be stored and loaded (the program allows the information to be saved when the series is finished). The operator can save all information appearing on the screen while the work is in progress.

*Option 5: Graphic presentation*

This option permits peak-to-peak presentation, elimination of non-desired peaks, smoothing operations (elimination of spurious signals on a peak, standardization of the base-line) and can show the distribution of homogeneous peaks on the time axis. The option provides a much better data presentation than that produced in routine work.

*Reagents and apparatus*

The electronic device and the behaviour of the program, especially when data are obtained from different instruments, were tested with the department's research work. The stock solutions and apparatus used were: aqueous solutions of potassium permanganate (Merck, a.r.) used in spectrophotometric readings with the aid of a CE

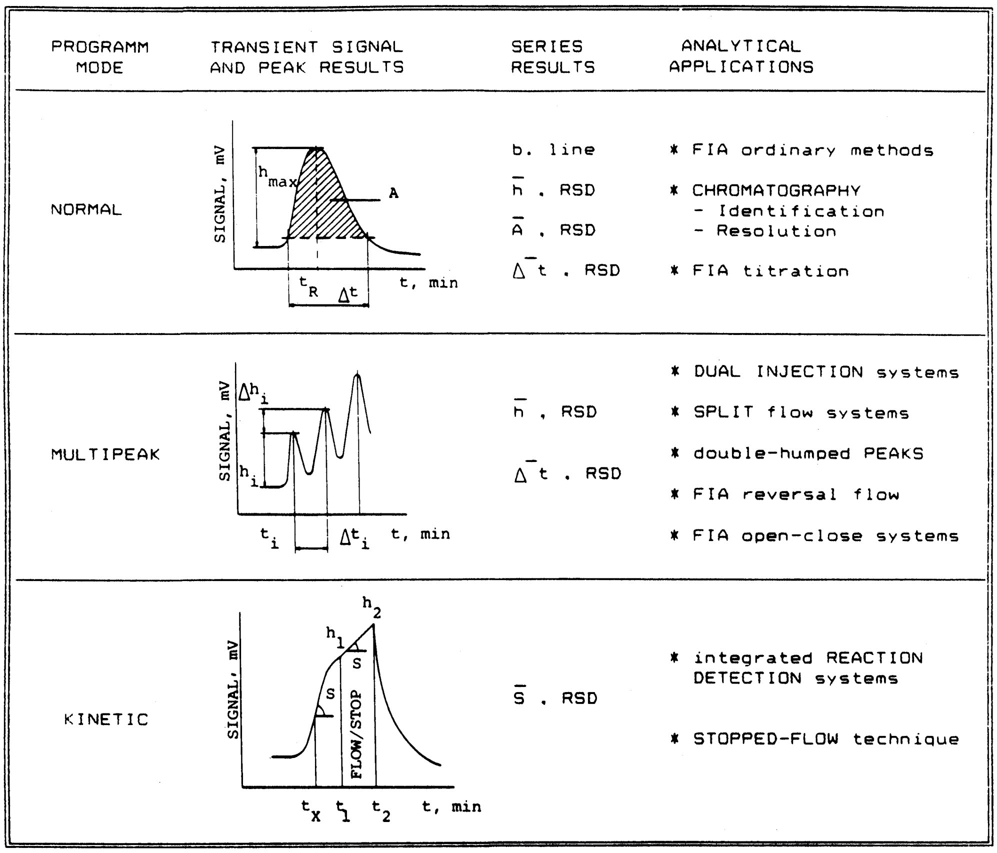

*Figure 3. Main features, results and applications of the device and the proposed program.*

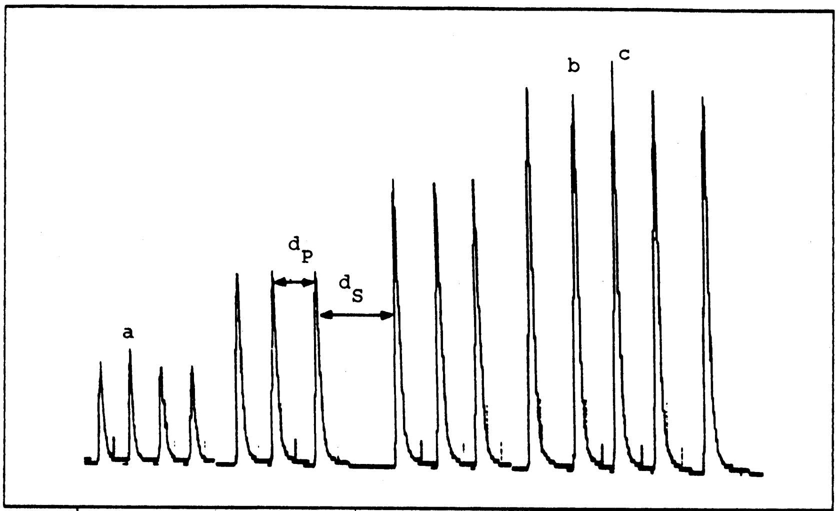

| PEAKS | 1 2 3 4 | 1 2 3 | 1 2 3 | 1 2 3 4 5 |
|---|---|---|---|---|
| SERIES | 1 | 2 | 3 | 4 |
| SELECTED PEAKS | 1,3,4 | 1,2,3 | 1,2,3 | 1,4,5 |

*Figure 4. Nomenclature for the transient signals and option features (see text for details).*

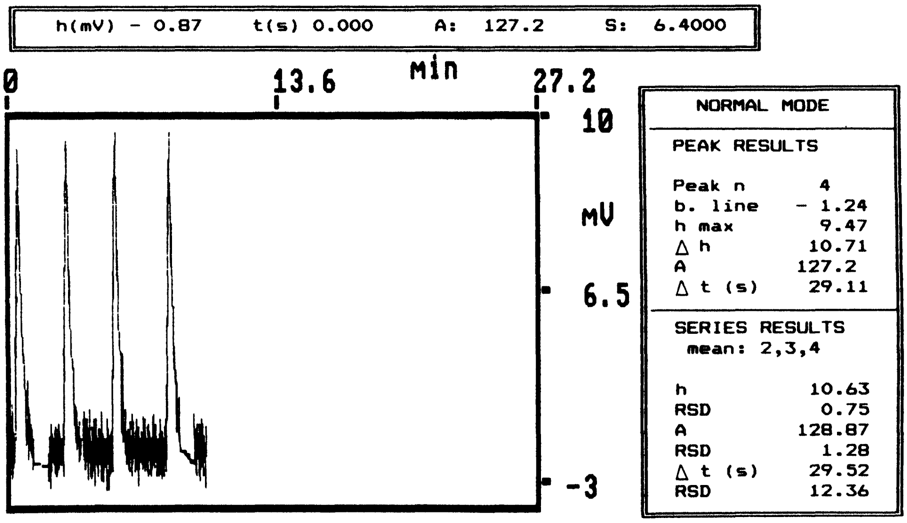

*Figure 5. Screen showing the Normal mode application (see text for details).*

spectrophotometer (Cecil Instruments), aqueous solution of diphenhydramine hydrochloride (Sigma, pure) and ammonium Ce(IV) nitrate (Merck, a.r.) used for fluorometric measurements with a Simadzu RF-250 spectrofluorimeter. Atomic absorption peaks were obtained from injections of glycine (Probus, a.r.) plus sorbitol (Acofarma, puro) as interference, passing through a fine powdered $CuCO_3$ $Cu(OH)_2$. 10 $H_2O$ (Probus, a.r.) column. The instrument used was a SP 1900 AAS from Unicam. Finally, turbidimetric signals from chlorhexidine hydrochloride injections, using the CE 202 spectrophotometer, and electroanalytical readings of Cu(II), obtained with a micropH 2002 (a Crison selective electrode ion-potentiometer), have been also performed successfully.

The FIA assembly used in all cases was a manifold formed by a Minipuls 2 (Gilson) peristaltic pump; a 80 μl flow-cell (spectrophotometric measurements) and a 30 μl flow-cell (fluorometric measurements), (both from Hellma); a Rheodyne 5041 injection valve; and 0·5 mm internal diameter Teflon tube coils. For atomic absorption, a HPLC Shimadzu/LC-GA pump was used.

### Results and discussion

Real-time data are very useful for studies on transient signal evolution, and they can help with theoretical studies on chemical and physical aspects of flow systems. The presentation of the slope and the partial peak area during the peak appearance are generally inadequate in real time. Figure 3 indicates the results provided by the program after the peak and series of peaks finalization, from the three modes of operation. Figure 3 also shows the different kinds of transient signals that can be treated, as well as their application. These signals cover the main needs for routine and research work in FIA analyses. Simple calculations from data provided by the program could satisfy needs in chromatography.

As an example of the capabilities of the different software options, as well as the nomenclature and the peak and series number assignation, figure 4 shows a calibration graph obtained by the injection of four solutions of potassium permanganate. The program was running in the normal mode and using the standard conditions

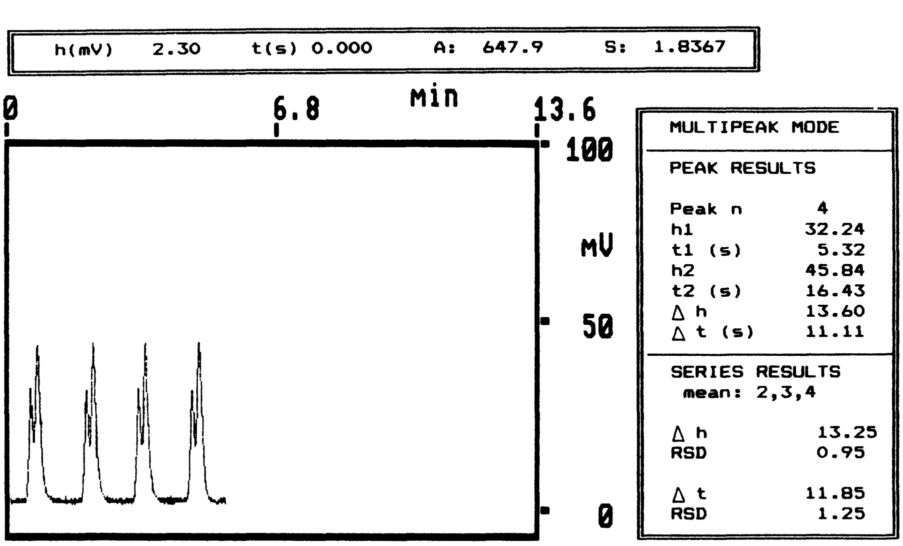

*Figure 6. Screen showing the Multipeak mode application (see text for details).*

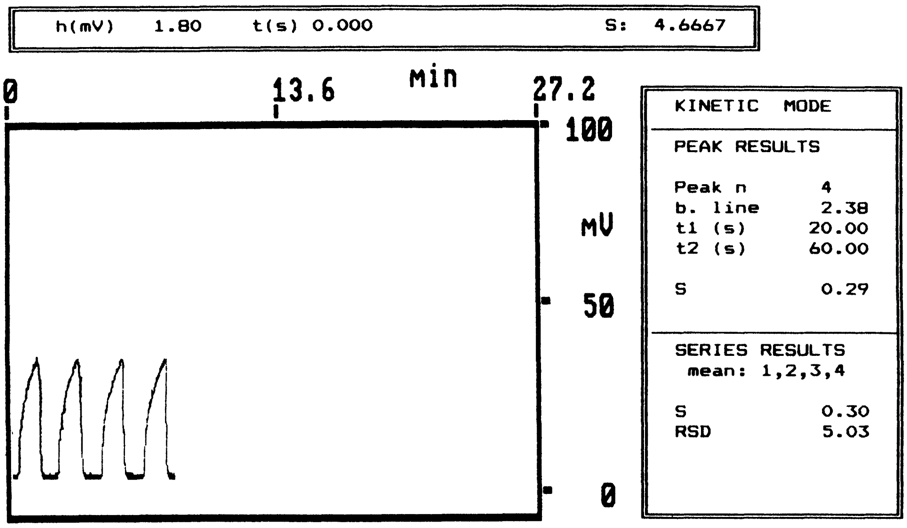

*Figure 7. Screen showing the Kinetic mode application (see text for details).*

(three peaks/series and RSD value up 1% for the peak-height in mV). In this example, the second peak from the first series (marked 'a' in this figure), was eliminated because the RSD value exceeded 1% when the first three peaks were obtained, the second one being the worst. A fourth peak was then included. The final series results (average signal, area and time-width) were then calculated for peaks 1, 3 and 4. The same problem occurred in the fourth series when peaks 1, 4 and 5 were selected. This will be of interest when automatic control of a large number of samples is required – this option could then be used as the criterion followed by the autosampler unit to change the sample.

A well-known advantage of an electronic system for the measurement of data, over the use of a manual method for peak-height calculation, is the high accuracy and sensitivity obtained (here the peak-height is given at ±0·01 mV level). From the experience in figure 4, after the peak-selection step, the correlation coefficient value was 0·999998, confirming the good performance of the acquisition–calculation procedure. If desired the presentation of the preceding peaks can be examined and then automatically re-scanned by the program, using option 5 from the main menu; i.e. peaks not selected 'a', 'b', 'c', may be eliminated, distance peak to peak, '$d_p$', and distance series to series, '$d_s$', may be equilibrated, the noise showed by the base-line can be erased etc.

Some practical examples, from the current work in this laboratory, have been used to test the device and the program. Three examples were selected, which corresponded to three modes: Normal, Multipeak and Kinetic. Figures 5, 6 and 7 have been taken from the computer; the original Spanish has been translated. The location of results on the screen is the same in each case, however, it is different for each mode, as has been indicated in figure 3. Real-time data are continuously shown on the upper half of the screen. Peak-to-peak and final series results appear on the right-hand side of the screen.

Figure 5 shows the screen when running routine FIA experimental work (followed by the NORMAL mode). The peak-height, in mV, is the analytical signal selected for evaluation. The peaks are obtained from consecutive injections of a glycine solution containing sorbitol (as an interferent to its determination). The carrier-reagent solution was an aqueous $HCO_3^-/CO_3^{2-}$ solution of pH 9·5 and was pumped by an HPLC pump through a mini-reactor filled with a copper basic carbonate. When a Cu(II)-glycine complex was formed, the transient signals (due to the Cu(II) and proportional to glycine concentration) were monitored by means of an atomic absorption instrument [15], and then treated by the authors' device. It is important to note the shortness of the scale, which produces a high level of sensitivity.

The fluorescence of the Ce(III) formed after the redox reaction when an aqueous solution of Diphenhydramine was injected into a carrier-reagent stream containing Ce(IV) in diluted sulphuric acid medium [16], was used to apply the Multipeak mode and the stopped-flow strategy using the Kinetic mode. Results are shown in figures 6 and 7 respectively. Using the Multipeak mode, the number of peaks per injection to be considered has to be previous introduced in the computer (two peaks in the present example, figure 6). In the last case (figure 7), the pump is stopped 20 s after the injection and switched-on again at 60 s.

### Conclusions

The main advantage of using classical transductors as chart recorders for data presentation is versatility. Data evaluation is, however, very limited. State of the art analytical instrumentation is now computerized and allows sophisticated treatment and graphing; however, the data-acquisition systems available do not give the operator access to the software. A home-made device, such as the one described here, can offer the advantages of the two strategies, as well as solving their problems. The device can offer general and specialized use and because of the low cost of electronic components and computers, it is an inexpensive option.

### References

1. Betteridge, D. and Goad, T. B., *Analyst*, **106** (1981), 257.
2. *Talanta*, **28** (1981), 7B.
3. Gisin, M. and Thommen, C., *Trends in Analytical Chemistry*, **8**(2) (1989), 62.
4. Bauer, W. E., Wade, A. P. and Crouch, S. R., *Analytical Chemistry*, **60** (1988), 287.
5. Papamichel, E. and Evmiridis, M. P., *Trends in Analytical Chemistry*, **7**(10) (1988), 366.
6. Egan, R. E. and Huber, J. W., *Am. Lab.*, **20** (1988), 22.
7. Fasanmade, A. A. and Fell, A. F., *Analytical Chemistry*, **61** (1989), 720.
8. Rittenhouse, R. C., *Journal of Chemical Education: Software*, **1B**(2) (1989), 17.
9. Hodges, R. S., Robert Parker, J. M., Mant, C. T. and Sharma, R. S., *Journal of Chromatography*, **458** (1988), 147.
10. Goulder, D., Blaffert, T., Blokland, A., Buydnes, L., Chabra, A., Cleland, A., Dunand, N., Hindriks, H., Kateman, G. *et al., Chromatographia*, **26** (1988), 237.
11. De Smet, M., Peeters, A., Buydens, L. and Massart, D. L., *Journal of Chromatography*, **457** (1988), 25.
12. Peichang, L. and Hongxin, H., *Journal of Chromatography*, **452** (1988), 175.
13. Luque de Castro, M. D. and Valcarcel, M., *Trends in Analytical Chemistry*, **8**(5) (1989), 172.
14. Luque de Castro, M. D. and Valcarcel, M., *II International Symposium on Pharmaceutical and Biomedical Analysis*, York (UK), (April 1990).
15. Martínez Calatayud, J. and Mateo García, J. V., *II International Symposium on Pharmaceutical and Biomedical Analysis*, York (UK), (April 1990).
16. Martinez Calatayud, J., Sagrado Vives, S. and Blasco Martinez, J., *II International Symposium on Pharmaceutical and Biomedical Analysis*, York (UK), (April 1990).

